# Activation of Aspen Wood with Carbon Dioxide and Phosphoric Acid for Removal of Total Organic Carbon from Oil Sands Produced Water: Increasing the Yield with Bio-Oil Recycling

**DOI:** 10.3390/ma9010020

**Published:** 2016-01-02

**Authors:** Andrei Veksha, Tazul I. Bhuiyan, Josephine M. Hill

**Affiliations:** 1 Department of Chemical and Petroleum Engineering, University of Calgary, 2500 University Drive NW, Calgary, AB T2N 1N4, Canada; tibhuiya@ucalgary.ca; 2 Residues and Resource Reclamation Centre, Nanyang Environment and Water Research Institute, Nanyang Technological University, 1 Cleantech Loop, Clean Tech One, Singapore 637141, Singapore; aveksha@ntu.edu.sg

**Keywords:** activated carbon, adsorption, bio-oil, pore volume, water treatment, yield

## Abstract

Several samples of activated carbon were prepared by physical (CO_2_) and chemical (H_3_PO_4_) activation of aspen wood and tested for the adsorption of organic compounds from water generated during the recovery of bitumen using steam assisted gravity drainage. Total organic carbon removal by the carbon samples increased proportionally with total pore volume as determined from N_2_ adsorption isotherms at −196 °C. The activated carbon produced by CO_2_ activation had similar removal levels for total organic carbon from the water (up to 70%) to those samples activated with H_3_PO_4_, but lower yields, due to losses during pyrolysis and activation. A method to increase the yield when using CO_2_ activation was proposed and consisted of recycling bio-oil produced from previous runs to the aspen wood feed, followed by either KOH addition (0.48%) or air pretreatment (220 °C for 3 h) before pyrolysis and activation. By recycling the bio-oil, the yield of CO_2_ activated carbon (after air pretreatment of the mixture) was increased by a factor of 1.3. Due to the higher carbon yield, the corresponding total organic carbon removal, per mass of wood feed, increased by a factor of 1.2 thus improving the overall process efficiency.

## 1. Introduction

Activated carbon from wood is widely used as an adsorbent for water treatment—specifically to adsorb organic compounds. As reported previously, the adsorption capacity of carbon depends on the properties of the adsorbing molecules (e.g., molecular size, structure, solubility and pK_a_) and adsorption conditions (e.g., pH, ionic strength and temperature) [1]. Moreover, the adsorption capacity is influenced by the properties of the carbon itself, including porosity (pore volume and pore size distribution), surface functionalities (e.g., oxygen and nitrogen groups) and mineral matter content [1,2,3,4].

Both physical and chemical activation techniques have been applied to produce activated carbon for water treatment. Preparation by physical activation consists of pyrolysis of the raw material (*i.e.*, heating in an oxygen deprived atmosphere), and results in a solid carbon (char), which can be further treated (*i.e.*, activated) at high temperature with oxidizing agents, such as steam, CO_2_ or air. Alternatively, activated carbon can be produced by chemical activation, in which the feedstock is impregnated with KOH, H_3_PO_4_, ZnCl_2_, FeCl_3_, *etc*. The removal of organic compounds from water by carbon samples with different properties (*i.e.*, produced with different activation methods) was compared previously [5,6,7,8]. Okada *et al*. [5] prepared carbon by activation of waste newspaper with K_2_CO_3_ and steam, and showed that K_2_CO_3_ activated carbon had higher methylene blue uptake due to larger surface area, pore sizes and higher content of surface oxygen groups. Girgis *et al*. [6] compared the adsorption of methylene blue by activated carbon from peanut hulls prepared by H_3_PO_4_, ZnCl_2_, KOH and steam activation, and found the largest adsorption capacities for the H_3_PO_4_ activated carbon, while KOH activated carbon had the lowest uptake. In a recent study [7], naphthenic acid removal from water was higher on wood activated by H_3_PO_4_ than by CO_2_, probably due to the larger mesopore volumes and surface areas obtained with H_3_PO_4_. Mestre *et al*. [8], investigated the adsorption of pharmaceutical compounds by activated carbon from cork prepared with steam, KOH and K_2_CO_3_. The uptakes of ibuprophen, paracetamol, acetylsalicylic acid, caffeine, and clofibric acid were higher by chemically activated carbon, while the uptake of iopamidol was higher by physically activated carbon. These results were attributed to the differences in micro and mesopore structures of the activated carbon.

Cost analysis of carbon production by physical (steam and CO_2_) and chemical (H_3_PO_4_) activation of almond shells, pecan shells and vetiver roots has been performed [9,10,11,12], the large capital and reactant investments for H_3_PO_4_ activation were offset by high activated carbon yields. In contrast, the capital and operational expenses for physical activation are lower but the yields are also lower. The production costs of activated carbon for these materials were estimated as 1.46–2.72 $·kg^−1^ [10,11,12], 2.56 $·kg^−1^ [10] and 1.17–2.89 $·kg^−1^ [9,11,12] for steam, CO_2_ and H_3_PO_4_ activation, respectively. Low yields of physically activated carbon are attributed to carbon loss during both the pyrolysis and activation stages. Depending on the raw material and operating conditions, only 43%–63% of carbon in biomass is converted to char during pyrolysis while the remaining carbon is contained in bio-oil and pyrolysis gases [13]. To some extent, char yield can be improved by optimizing reaction conditions, such as particle size, moisture content in feedstock, pressure and residence time of volatile pyrolysis products in the reactor as reviewed by Antal and Gronli [14]. Activated carbon yield can also be increased by reducing carbon loss during the activation stage. For this purpose, air gasification followed by carbonization in an inert atmosphere to decompose chemisorbed oxygen has been proposed [15,16,17]. Although this approach increased the surface area, multiple heat treatment steps are required to produce the activated carbon.

As stated above, the efficacy of activated carbon as an adsorbent for organic compounds is partially determined by its properties and these properties can be manipulated by the activation method [5,6,7,8]. Thus, one of the objectives of this study was to investigate which activation method is suitable for the preparation of carbon specifically for the removal of total organic carbon (TOC) from oil sands produced water (specifically from steam assisted gravity drainage, SAGD). SAGD is a common method for bitumen extraction from oil sands. In this technique, two parallel wells are drilled horizontally into the oil sands deposit. Steam generated at a surface plant is pumped into the deposit through the top well to soften the bitumen. The bitumen and condensed steam are then pumped to the surface from the bottom well for processing and separation. The separated water, so called oil sands produced water, contains high concentrations of water soluble organic compounds. In particular, the SAGD water used in this study had a total organic carbon content of ~1000 mg·L^−1^. Because ~90% of this water is recycled, the presence of organic compounds can cause both corrosion and fouling on equipment that result in periodic shutdowns for cleaning and increase operating costs [18]. The activated carbon samples were prepared from locally available aspen wood residues, thus lowering the environmental impact from transportation. CO_2_ (physical) and H_3_PO_4_ (chemical) activation were used and the yield, TOC removal, and, ultimately, the process efficiency of both methods determined. In addition to as prepared activated carbon, acid washed and heat treated samples with reduced ash content and surface functionalities were prepared to determine the effect of these parameters [1,2,3,4], if any, on the TOC uptake and clarify the role of porosity.

The second objective of the current study was the investigation of a new method to increase the yield of activated carbon prepared by physical activation. This method is based on the recycling of bio-oil from previous runs to the raw feedstock (*i.e.*, biomass) for pyrolysis in order to increase char production. Bio-oil from biomass is a carbon-rich product with limited application due to acidity, corrosiveness, high oxygen and moisture content, and coking upon reheating [19]. Previous studies have shown that depending on the raw material and operating conditions, the addition of bio-oil to biomass increases the char production by 10%–43% [20,21,22,23]. Although the char yield was increased, it is not obvious how the deposited and subsequently pyrolyzed bio-oil will impact the final activated carbon product. Thus, this study included the investigation of the CO_2_ activation of char produced from a mixture of biomass and bio-oil. The influence of the char preparation conditions on yield, porosity and removal of organic compounds from SAGD water are discussed.

## 2. Results and Discussion

### 2.1. TOC Removal by Activated Carbon from Wood

Activated carbon samples prepared with CO_2_ and H_3_PO_4_ activation of aspen wood were used to investigate the influence of activation method on the TOC removal from SAGD water. The development of porous properties in the activated carbon samples during CO_2_ (at 800 °C) and H_3_PO_4_ (at 500 °C) activation were controlled by activation time and H_3_PO_4_:wood ratio, respectively. After H_3_PO_4_ activation, the acid residue was removed by washing with deionized water. In addition to as prepared activated carbon samples, acid washed and heat treated (at 900 °C for 1 h in nitrogen) samples with reduced ash content and surface functionalities were prepared to minimize their effect, if any, on the TOC uptake and clarify the role of porosity. The samples prepared by CO_2_ activation are denoted as AC—for activated carbon—followed by the activation time in hours. Samples activated with H_3_PO_4_ were denoted by the letter P followed by the H_3_PO_4_:wood ratio of 1:2, 1:1 or 2:1. The abbreviation “HT” denotes samples that were acid washed and heat treated (HT). The TOC removal by the activated carbon samples was compared with a non-activated char (Char-800-HT) prepared from the same feedstock (at 800 °C) and commercial activated carbon (as supplied ColorSorb and acid washed and heat treated ColorSorb-HT) specially designed for the removal of organic molecules and decolorization.

The UV-vis spectrum of the SAGD water in Figure S1 demonstrates light absorbance between 200 and 500 nm, with increasing absorbance at lower wavelength. There was no distinct peak at 254 nm on the spectrum, representative of aromatic compounds. The possible reason is interference from non-aromatic molecules (acetone, 2-butanone, naphthenic acids) contained in large quantities in the water [24].

**Figure 1:**
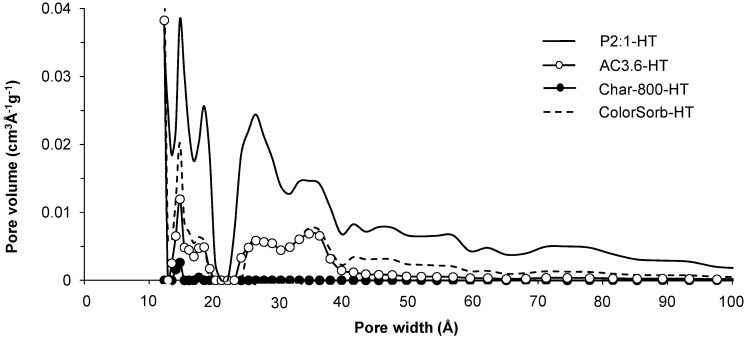
Pore size distributions of selected activated carbon determined with non-local density functional theory.

The porous properties of the samples are listed in Table 1 and N_2_ adsorption and desorption isotherms of the selected samples are shown in Figure S2. An increase in CO_2_ activation time resulted in increased surface areas and micropore volumes determined by N_2_ adsorption (pore sizes up to 20 Å) from 540 m^2^·g^−1^ and 0.18 cm^3^·g^−1^ for 0.3 h treatment (AC0.3-HT) to 910 m^2^·g^−1^ and 0.30 cm^3^·g^−1^ for 3.6 h treatment (AC3.6-HT). The micropore volumes determined by CO_2_ (pore sizes less than ~7 Å) also increased with activation time from 0.23 to 0.32 cm^3^·g^−1^. The meso/macropore volume increased during the first hour of activation up to 0.22 cm^3^·g^−1^ and did not change significantly thereafter. As expected, the surface areas and pore volumes of CO_2_ activated carbon samples were higher compared to non-activated char (Char-800-HT). With H_3_PO_4_ activation, higher H_3_PO_4_:wood ratios resulted in higher surface areas and meso/macropore volumes, while the micropore volume determined by N_2_ adsorption was the largest in the sample produced with a H_3_PO_4_:wood ratio of 1:1. The micropore volume determined by CO_2_ adsorption did not change with an increase in H_3_PO_4_ concentration. Compared to CO_2_ activation, H_3_PO_4_ activation generally resulted in larger surface areas and micropore volumes (determined by N_2_ adsorption). Pore size distributions of four of the activated carbon samples are shown in Figure 1. H_3_PO_4_ activation produced the widest pore diameters. More specifically, the P2:1-HT sample contained pores with diameters up to 100 Å, while the pores in samples AC3.6-HT and ColorSorb-HT were smaller than 40 Å and 60 Å, respectively. In the non-activated Char-800-HT sample no pores larger than 15 Å were observed.

**Table 1:** Properties of activated carbon prepared by physical and chemical activation of wood, with and without heat treatment.

Sample	Yield (%)	Meso/Macropore Volume-N_2_ (cm^3^·g^−1^)	Micropore Volume-N_2_ (cm^3^·g^−1^)	Micropore Volume-CO_2_ (cm^3^·g^−1^)	BET-N_2_ Surface Area (m^2^·g^−1^)
**Char**					
Char-800-HT	21	0.02	0.17	0.23	440
**CO_2_ Activated Carbon**					
AC0.3-HT	19	0.09	0.18	0.23	540
AC1.0-HT	15 ± 1 ^a^	0.22	0.19	0.28	690
AC1.0		0.21	0.16	0.23	600
AC1.8-HT	11	0.21	0.22	0.27	750
AC3.6-HT	6	0.19	0.30	0.32	910
**H_3_PO_4_ Activated Carbon**					
P1:2-HT	46	0.04	0.35	0.27	870
P1:1-HT	45	0.11	0.46	0.26	1110
P1:1		0.09	0.60	0.24	1240
P2:1-HT	41	0.71	0.40	0.26	1350
**Steam Activated Commercial Carbon**					
ColorSorb-HT	-	0.30	0.36	0.37	1140
ColorSorb		0.31	0.30	0.32	988

^a^ Average of six measurements ± standard deviation.

**Figure 2:**
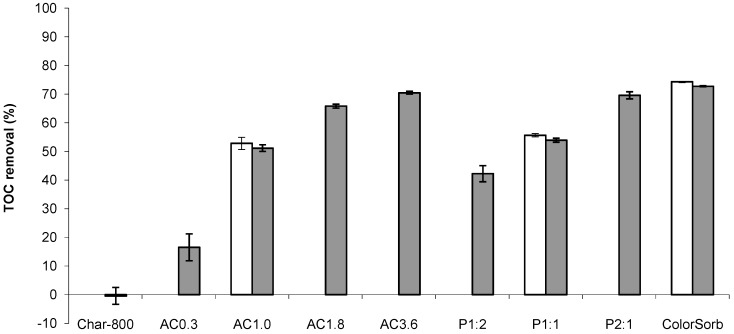
Total organic carbon (TOC) removal by activated carbon from steam assisted gravity drainage (SAGD) water (white and grey bars for as prepared and heat treated (HT) carbon, respectively).

The percentages of TOC removed from SAGD water by the prepared activated carbon samples are shown in Figure 2. The TOC uptakes by the heat treated samples (AC1.0-HT, P1:1-HT, ColorSorb-HT) were similar to those by the corresponding as prepared samples (AC1.0, P1:1, ColorSorb), suggesting that ash components and surface functionalities removed from the carbon by acid washing and heat treatment, respectively, had little if any influence on the TOC removal. To investigate whether the sample acidity/basicity could influence the TOC uptake, the pH of the water was measured after adsorption. Compared to the feed SAGD water with pH 9.5–9.8, the pH after adsorption on all activated carbon samples did not change more than 0.7 units. Figure S3 shows the influence of pH on the precipitation of TOC. There are no changes in TOC concentration till pH 7 followed by a rapid decrease in TOC content in water at pH below 7. These data suggest that to influence water chemistry and, consequently, adsorption behavior by the samples, a decrease in pH by at least ~2.5–2.8 units from the initial value is required, which is larger than that observed for all samples.

Longer activation times with CO_2_ and higher H_3_PO_4_:wood ratios increased the TOC removal, which could be attributed to the development of porosity during activation as discussed below. In contrast, non-activated Char-800-HT did not remove organic compounds from SAGD water, suggesting that activation was a necessary step for the development of the pores responsible for adsorption. These results are consistent with a previous study [25], in which non-activated char was found to be ineffective for the removal of methyl orange from aqueous solutions. Regardless of the activation process, the maximum TOC removal by the prepared activated carbon samples was ~70%, which was similar to the removal by the commercial steam activated carbon from wood (*i.e.*, ColorSorb).

**Figure 3:**
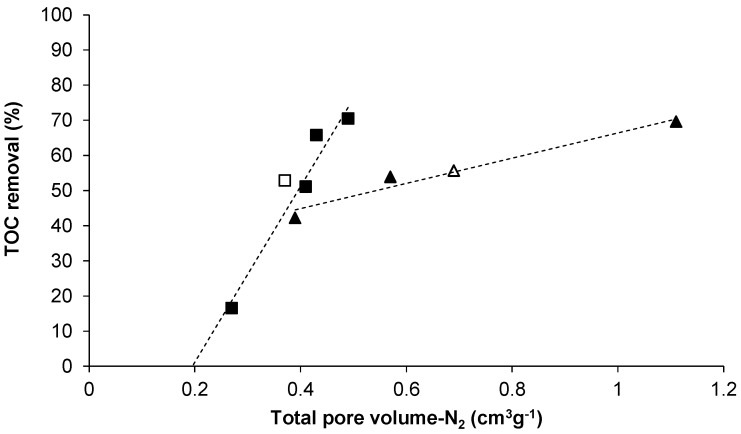
Relationship between TOC removal and total pore volumes of the activated carbon: open and closed squares for as prepared and heat treated CO_2_ activated carbon, respectively; open and closed triangles for as prepared and heat treated H_3_PO_4_ activated carbon, respectively.

It is hard to define the range of pore sizes responsible for the removal of TOC by activated carbon, as SAGD water contains a large number of organic compounds with various sizes and chemical structures [24,26]. However, it is likely that micropores probed by CO_2_ (smaller than 7 Å) did not contribute to the adsorption of TOC, as these pores were present in all carbon samples, including the non-activated carbon (Char-800-HT) that had no TOC removal (Figure 2). In contrast, pores probed by N_2_ were suitable for TOC removal as suggested by Figure 3 in which the TOC uptake is plotted versus total pore volume. The TOC removal increased with total pore volume for the samples activated with either CO_2_ or H_3_PO_4_. The pore volume was increased by activating for longer times or increasing the H_3_PO_4_:wood ratio (Table 1). According to Figure 3, the data sets for CO_2_ and H_3_PO_4_ activated carbon samples have different slopes, which could be due to either different mechanisms of TOC uptake by the samples or due to removal of different compounds from the water. A more detailed study is required to clarify this phenomenon. Nevertheless, from a practical viewpoint, the linear relationship between TOC removal and total pore volume suggests that the latter can be utilized as a screening parameter during selection of activated carbon prepared by the same activation method for SAGD water treatment.

The TOC removal was comparable for CO_2_ and H_3_PO_4_ activation (Figure 2) but the yields weredifferent—less than 20% for the former compared to greater than 41% for the latter (Table 1). These yields translate to 4.8–17.5 g of wood consumed per 1 g of CO_2_ activated carbon and only 2.2–2.4 g of wood consumed per 1 g of H_3_PO_4_ activated carbon. The low yield of CO_2_ activated carbon is attributed to the significant mass loss during pyrolysis. Besides conversion to char, wood components, such as hemicellulose, cellulose and lignin, undergo depolymerization, fragmentation and conversion to volatile pyrolysis species upon heating as reviewed by Di Blasi [27]. Although some of the volatile species can be partially converted to char by the secondary reactions, they mainly form condensable bio-oil and non-condensable gases [13,22,28]. Further losses occur during activation from the partial gasification of the char by the oxidizing agent (in this study CO_2_) to develop porosity. In the case of H_3_PO_4_ activation, higher yields of carbon from lignocellulosic materials, including wood, are attributed to crosslinking reactions between decomposing fragments via phosphate and polyphosphate bridges that prevent release of relatively small molecules from the solid phase [29]. After the removal of (poly)phosphate bridges by water washing, the structure of the produced carbon remains in an expanded state with an accessible pore structure [29].

### 2.2. Increasing the Yield of CO_2_ Activated Carbon

**Figure 4:**
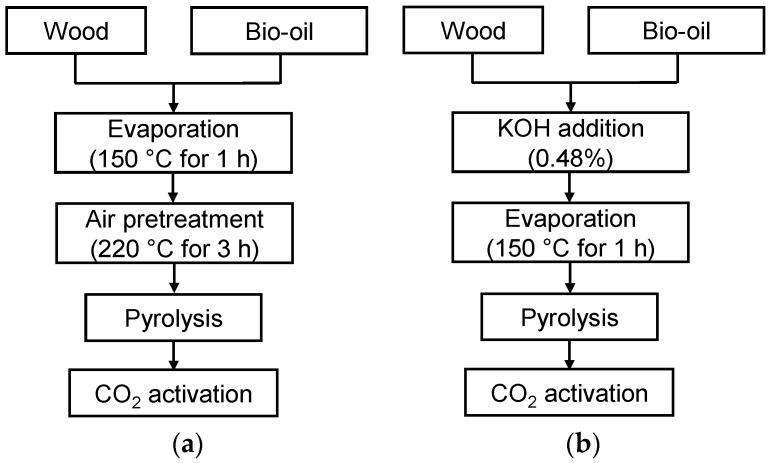
Activated carbon preparation methods with air pretreatment (**a**) and KOH addition (**b**) prior to pyrolysis of wood with bio-oil.

A method based on the recycling of bio-oil from previous runs [23] will now be used to increase the yield of activated carbon. In the results presented thus far, the feed was only wood. Subsequent results are those obtained with a feed of wood and bio-oil. Figure 4 illustrates the two approaches used to prepare porous carbon samples by CO_2_ activation. In the first approach, the mixture of wood and bio-oil undergoes air pretreatment at 220 °C for 3 h in order to stabilize the mixture and then the temperature is increased up to 600 °C for the pyrolysis (Figure 4a). A 3 h pretreatment with air at 220 °C is sufficient to achieve the maximum char mass gain [23]. In the second approach, 0.48% KOH is added to the mixture of wood and bio-oil to catalyze char formation upon heating to 600 °C (Figure 4b). The content of KOH was limited to 0.48% to keep the ash content in the char at ~5%, which is considered a maximum for a good quality product [14]. Specifically, ash contents in the char from wood, mixture of wood and bio-oil with air pretreatment and KOH addition were 2.0%, 1.4% and 5.5%, respectively. Mineral matter/ash can block the pores and preferentially adsorb water, hindering adsorption of the desired adsorbate [1]. Previously, it was demonstrated that air pretreatment and KOH addition prior to pyrolysis result in the higher char yields from wood and bio-oil mixture compared to the pyrolysis without these treatments [23].

In both approaches water and organic compounds with boiling points below 150 °C were removed by evaporation at 150 °C (Figure 4). Otherwise, these compounds accumulate in bio-oil upon continuous recycling to new batches of wood. Similar to the AC1.0 sample, the char samples produced from the mixture of wood and bio-oil were activated with CO_2_ for 1 h at 800 °C. The char samples prepared by KOH addition and air pretreatment of wood and bio-oil mixture are denoted as Char-600-KOH and Char-600-Air, respectively. The activated carbon prepared with KOH addition to the wood and bio-oil mixture includes “KOH” in the name followed by HT, if applicable, (e.g., AC1.0-KOH-HT is activated carbon prepared from wood with bio-oil and KOH by CO_2_ activation for 1 h followed by acid washing and heat treatment), while the carbon prepared by air pretreatment of wood and bio-oil includes “Air” in the name.

Table 2 lists the properties of the parent char samples and the CO_2_ activated carbon. The char yields produced by bio-oil recycling increased to 30.4% and 30.9% (Char-600-Air and Char-600-KOH, respectively) compared to 23.7% for the Char-600 sample. The porous properties of Char-600-Air were similar to Char-600, while the porosity of Char-600-KOH was lower, likely due to pore blockage by potassium compounds as discussed in our previous study [23]. After activation, the yield of the AC1.0-Air carbon was 19.4% (Table 2), which is 1.3 times larger than the yield of AC1.0 from wood (15%, Table 1). In contrast, despite the increased yield of Char-600-KOH (30.9%), the yield of AC1.0-KOH was only 15.4% after activation, suggesting that the addition of potassium hydroxide for the pyrolysis of wood with bio-oil was not beneficial for the production of activated carbon under these conditions. The lower yield of this activated carbon sample compared to AC1.0-Air can be attributed to the enhanced carbon loss due to the reaction with CO_2_ in the presence of potassium compounds. Potassium compounds are known catalysts for char gasification by CO_2_ [30].

The acid washing and heat treatment of activated carbon prepared from the mixture of wood and bio-oil significantly increased the porosity (Table 2), in contrast to the minor improvement achieved for sample AC1.0 (compare to AC1.0-HT in Table 1). In the AC1.0-KOH-HT carbon, the observed increase in porosity could be partially explained by the presence of potassium compounds, which can enhance the development of pores during CO_2_ activation [31]. Total pore volume of as prepared AC1.0-KOH calculated on an ash free basis was larger than those of AC1.0 and AC1.0-Air samples (0.43 cm^3^·g^−1^, 0.38 cm^3^·g^−1^ and 0.35 cm^3^·g^−1^, respectively). However, the increase in porosity was also observed in AC1.0-Air-HT, prepared without potassium hydroxide addition (Table 2). Therefore, it is likely that the observed phenomenon was primarily attributed to the use of bio-oil for the preparation of the activated carbon rather than to KOH.

**Table 2:** Yield and porous properties of carbon produced from wood and bio-oil mixture.

Sample	Yield (%) ^a^	Meso/Macropore Volume-N_2_ (cm^3^·g^−1^)	Micropore Volume-N_2_ (cm^3^·g^−1^)	Micropore Volume-CO_2_ (cm^3^·g^−1^)	BET-N_2_ Surface Area (m^2^·g^−1^)
**Char from Wood**					
Char-600	23.7 ± 0.4 ^b^	0.02 ^b^	0.17 ^b^	0.18 ^b^	440 ^b^
**Char from Wood and Bio-Oil Mixture**					
Char-600-Air	30.4 ± 0.7	0.03	0.17	0.20	450
Char-600-KOH	30.9 ± 0.1	0.02	0.01	0.15	30
**Activated Carbon from Wood and Bio-Oil Mixture**					
AC1.0-Air	19.4 ± 0.1	0.15	0.19	0.25	600
AC1.0-Air-HT		0.19	0.32	0.33	930
AC1.0-KOH	15.4 ± 0.1	0.20	0.18	0.24	640
AC1.0-KOH-HT		0.25	0.31	0.36	1020

^a^ Yield is calculated per mass of dried wood required for production of carbon based on the average of three measurements ± standard deviation; ^b^ data for Char-600 are from reference [23].

To investigate whether the bio-oil addition influenced the number of surface oxygen groups, the activated carbon prepared with air pretreatment was characterized with temperature-programmed decomposition (Figure S4). According to the literature [32,33,34], CO_2_ evolution can be attributed to decomposition of carboxylic groups and anhydrides (200–450 °C), peroxides (500–550 °C), lactols, lactones and anhydrides (above 600 °C). CO evolution can be ascribed to anhydrides (350–400 °C), phenols, hydroquinones (600–700 °C), carbonyls, quinones and ether groups (700–800 °C). At temperatures higher than 900 °C, CO evolves from pyrone and chromene groups. There were differences between CO and CO_2_ profiles of AC1.0-Air carbon from those of AC1.0 (Figure S4) due to different groups on the surfaces of the samples. However, the total amounts of CO_2_ and CO evolved from AC1.0-Air (19 µmol·g^−1^ and 114 µmol·g^−1^, respectively) were similar to those from AC1.0 (18 µmol·g^−1^ and 110 µmol·g^−1^ of CO_2_ and CO, respectively), suggesting no change in the number of surface oxygen groups due to bio-oil recycling.

**Figure 5:**
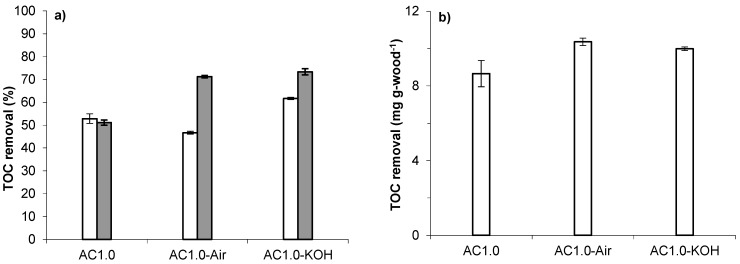
TOC removal by activated carbon prepared from the mixture of wood and bio-oil (white and grey bars for as prepared and HT carbon, respectively): (**a**) in per cent of removed organic carbon from water; and (**b**) in mg of organic carbon per mass of wood used for carbon production.

The porous carbon samples prepared from wood and bio-oil were then tested for TOC removal and the results are shown in Figure 5a. Relative to the removal achieved by sample AC1.0 (results reproduced from Figure 2 for ease of comparison), the percentages of TOC removal were 6% lower and 9% higher for the as prepared AC1.0-Air and AC1.0-KOH samples, respectively. The differences are consistent with the differences in the porosities of the samples (Table 1 and Table 2). Acid washing and heat treatment of these samples improved the TOC removal levels up to ~70% (Figure 5a), similar to that achieved with samples AC3.6-HT, P2:1-HT and ColorSorb-HT (Figure 2). Again the increase is consistent with the increase in porosities after the acid washing and heat treatment (Table 2). The pH of the water was measured after contact with the AC samples. In all cases, the pH was between 9.2 and 9.7, with measurement errors of 0.2–0.3 units.

The results for the as prepared carbon have been normalized for their different yields and TOC capacities by expressing the TOC removal in terms of mass of organic carbon removed per mass of wood used to prepare the activated carbon (mg·g-wood^−1^) as shown in Figure 5b. This normalization indicates that it is beneficial to recycle the bio-oil in terms of TOC removal per mass of wood feed. As mentioned in the Introduction, recycling also eliminates the problem of handling and disposal of the bio-oil. The results were similar for the two activated carbon preparation methods (Figure 4) with slightly more TOC removal obtained with the activated carbon prepared with air pretreatment (AC1.0-Air) than KOH addition (AC1.0-KOH), and with 1.2 times higher removal for AC1.0-Air compared to AC1.0, which was prepared from wood only. Thus, a higher conversion efficiency of the feed (wood) to an adsorbent for water treatment was obtained by bio-oil recycling.

## 3. Materials and Methods

### 3.1. Preparation of Activated Carbon

Aspen (*Populus tremuloides*) wood chips provided by Alberta-Pacific Forest Industries Inc. (Boyle, AB, Canada) were used as the feedstock. According to the proximate analysis, the wood chips contained 18.5% fixed carbon, 80.9% volatile matter and 0.6% ash on a dry basis. The contents of carbon, hydrogen and nitrogen were 48.6%, 6.0% and 0.6%, respectively, and were reported previously [23]. Activated carbon was prepared by several different methods. The first method consisted of wood pyrolysis to produce a char that was then activated in the presence of CO_2_. The second method involved pyrolyzing the wood with recycled bio-oil with either KOH addition or with air-pretreatment prior to CO_2_ activation, and the third method involved treatment of the wood with phosphoric acid (H_3_PO_4_).

Char production and CO_2_ activation were carried out in a vertical packed bed reactor setup described previously [22]. Approximately 7.5 g of the dried and crushed wood chips (particle diameter 0.3–2.0 mm and particle length 0.3–5.0 mm) were loaded into the reactor (bed volume 35 cm^3^). The reactor was heated at 4 °C·min^−1^ under N_2_ flow (space velocity 1 min^−1^) to 600 °C and pyrolyzed at this temperature and gas flow for 30 min to produce char (named Char-600). Bio-oil was collected in a flask attached to the outlet of the reactor. For the preparation of activated carbon in this study, the activation temperature was fixed at 800 °C, while the degree of activation and porosity were varied by activation time. Approximately 1.5 g of Char-600 was loaded into the reactor (bed volume 10 cm^3^), purged with N_2_ for 1 h and then heated at 10 °C·min^−1^ under this N_2_ flow to 800 °C. After 800 °C was reached, N_2_ was switched to CO_2_ (space velocity 10 min^−1^) for activation. The activation time with CO_2_ varied from 0.3 to 3.6 h. The char sample (Char-800) without CO_2_ activation was prepared by heating Char-600 in N_2_ flow for 1 h at 800 °C. All samples were crushed and sieved (US mesh 100) to obtain particle sizes less than 150 μm before further treatment, adsorption tests and/or characterization.

To reduce the amount of ash and surface oxygen groups, some of the samples were washed with hydrochloric acid and then heat treated in N_2_ flow at 900 °C (maximum safe temperature for operation of the setup). To remove acid soluble ash components, approximately 2 g of sample was soaked in 0.1 M hydrochloric acid for 18 h, washed with deionized water until pH 5–5.5 (*i.e.*, pH of deionized water) and then dried at 105 °C for 24 h. To remove surface oxygen groups, the dried sample (~1.5 g) was loaded into a quartz boat, which was then placed into a horizontal furnace, purged with N_2_ (100 cm^3^·min^−1^) for 1 h and then heated at 10 °C·min^−1^ under N_2_ flow to 900 °C and held at this temperature for 1 h. 

Wood impregnated with bio-oil generated in a previous pyrolysis run was also used as a precursor of activated carbon. Two methods were adopted from our previous work [23] to produce char from the mixture of biomass and bio-oil; namely, KOH addition and air pretreatment prior to the pyrolysis. For KOH addition, 0.068 g of KOH (85% pure, Alfa Aesar, Thermo Fisher Scientific Inc., Waltham, MA, USA) was dissolved in 0.1 g of deionized water followed by addition of 4.0 g of bio-oil produced from Char-600 pyrolysis (due to mass loss during transfer, the actual mass of bio-oil added to the wood was ~3.8 g and the resulting KOH content was 0.48% for the feed). The mixture was added to 7.5 g of wood, and the impregnated wood was loaded into the reactor, heated to 150 °C for 1 h in N_2_, to remove the water and light organic compounds, and then heated in N_2_ at the conditions used for the preparation of Char-600. The char sample was then activated with CO_2_ for 1 h at 800 °C following the procedure used for the activation of Char-600.

For experiments with air pretreatment, 3.8 g of bio-oil and 7.5 g of wood were mixed in a beaker and then loaded into the reactor. The reactor was heated at 4 °C·min^−1^ in air (space velocity 1 min^−1^) first to 150 °C for 1 h, to evaporate water and light organic compounds, and then to 220 °C for 3 h. After the air pretreatment, samples were purged with N_2_ (space velocity 1 min^−1^) for 0.5 h and heated in N_2_ at the conditions used for the preparation of Char-600.

To prepare H_3_PO_4_ activated carbon, dried wood chips were used as received. Approximately 60 g of wood chips were placed into a 1 dm^3^ beaker to which 300 cm^3^ of an aqueous solution of H_3_PO_4_ (85% pure, BDH Aristar, VWR International, Radnor, PA, USA) were added. The volume of solution was kept constant while the concentration of H_3_PO_4_ was varied to target H_3_PO_4_:wood ratios of 1:2, 1:1 and 2:1 by weight. The mixture was soaked for 1 d at ambient temperature (23 °C) and then water was evaporated from the surface of wood chips by heating at 100 °C on a hot plate. The prepared mixture of H_3_PO_4_ and wood was left for two more days at ambient temperature for impregnation and then dried in air at 110 °C overnight. Activation was carried out in a horizontal alumina reactor with an inner diameter of 8 cm. Approximately 30 g of the H_3_PO_4_:wood mixture was loaded into the alumina reactor and heated at a constant rate (4 °C·min^−1^) to 500 °C under N_2_ flow (space velocity 1 min^−1^). The temperature was maintained at 500 °C for 0.5 h and then the reactor was cooled under N_2_ to 70 °C for sample removal. The activated carbon samples were washed with deionized water until pH of 5–5.5, dried overnight at 110 °C and then crushed and sieved (US mesh 100) to obtain particles less than 150 µm. After washing, the samples were either used or subjected to a further heat treatment at 900 °C under N_2_ for 1 h.

A commercial activated carbon, ColorSorb G5 prepared by steam activation of wood was provided by Jacobi Carbons AB (Kalmar, Sweden).

### 3.2. Characterization of Char and Activated Carbon

Yields of char and CO_2_ activated carbon were calculated as mass of carbon after pyrolysis and activation, respectively, per mass of dried wood. The yield of H_3_PO_4_ activated carbon was calculated as mass of dried carbon, after H_3_PO_4_ removal by water washing, per mass of dried wood.

Activated carbon samples were characterized with N_2_ and CO_2_ adsorption measured at −196 °C and 0 °C, respectively (Tristar 3000, Micromeritics Instrument Co., Norcross, GA, USA). Surface areas were calculated from N_2_ adsorption isotherms using the BET method [35]. Samples (~0.05 g) were degassed at 300 °C under vacuum (100 mTorr) for 3 h before adsorption. The surface areas were determined from linear BET plots at the relative pressure ranges between 0.02 and 0.3, assuming a value of 0.164 nm^2^ for the cross-section of the N_2_ molecule. Micropore volumes were obtained from N_2_ and CO_2_ adsorption isotherms using the *t*-plot with Carbon black STSA reference isotherm and Dubinin-Radushkevich methods [36,37], respectively. Total pore volumes were determined by N_2_ adsorption at relative pressures of 0.96–0.97. The meso/macropore volume was calculated as the difference between total pore volume and micropore volume determined by N_2_ adsorption. The non-local density functional theory (NLDFT, Autosorp 1C, Quantachrome Instruments, Boynton Beach, FL, USA) [38] was used to calculate the pore size distributions of the activated carbon.

The surface oxygen groups were quantified by temperature-programmed decomposition. Approximately 10 mg of sample was placed in a thermogravimetric analyzer (Cahn Thermax 500 apparatus, Thermo Fisher Scientific Inc., Waltham, MA, USA) and heated in N_2_ at 5 °C·min^−1^ to 1000 °C. The evolution of CO_2_ and CO during heating was detected with an infrared gas analyzer (Uras 26, ABB AO2020, ABB Ltd., Zurich, Switzerland) and recorded.

### 3.3. Total Organic Carbon Removal from Water

Steam assisted gravity drainage (SAGD) water was collected from the process stream of an industrial SAGD facility (Athabasca region, AB, Canada) immediately following the water softening stage but prior to entering the boiler. This 80 °C water had a pH of 9.5–9.8 and total organic carbon (TOC) content ranging from 910 to 1162 mg·dm^−3^, depending on the sample. After cooling, the samples were shipped to the University of Calgary and stored at 4 °C.

The removal of TOC from SAGD water by activated carbon was evaluated by batch adsorption. In a glass vial, 0.1 g of activated carbon was mixed with 10 cm^3^ of SAGD water reheated to 80 °C. To measure the initial TOC content in the SAGD water, vials with 10 cm^3^ of the reheated water without carbon addition were prepared. The mixtures and pure SAGD water were shaken at 80 °C (typical temperature for field operations) and 175 rpm for 18 h before analysis. A series of kinetic measurements have been carried out, and 18 h treatment was sufficient to attain adsorption equilibrium on the carbon samples. After settling for 2–3 min, the supernatant (~9 cm^3^) was obtained with a syringe and filtered through a 0.45 μm nylon membrane filter (VWR International, Radnor, PA, USA). The cooled solutions were analyzed by a TOC analyzer (TOC-VCPN, Shimadzu Corp., Kyoto, Japan). To avoid premature precipitation of the organic acids in the SAGD water (Figure S3), acidification (by HCl addition) was done directly in the TOC analyzer. Three replicates with each adsorbent were prepared and the results are reported as averages ± standard deviation of three measurements. The statistical significance of differences between obtained results was determined using a two-tailed *t*-test with a confidence interval of 95%.

After TOC removal by the carbon samples, the pH of water was measured using a Lab 850 pH meter (Schott Instruments GmbH/SI Analytics GmbH, Mainz, Germany) with BlueLine 56 pH electrode (SI Analytics GmbH, Mainz, Germany).

## 4. Conclusions

Conversion of wood to activated carbon for industrial water treatment was investigated. Both chemically (H_3_PO_4_) and physically (CO_2_) activated carbon samples removed similar percentages of total organic carbon from SAGD water. As CO_2_ activated carbon had lower yields compared to carbon prepared with H_3_PO_4_, a method to increase the yield, and the conversion efficiency of wood by CO_2_ activation was investigated. Specifically, bio-oil was recycled and added to the wood feed before CO_2_ activation. Air pretreatment of the bio-oil and biomass mixture (220 °C for 3 h) was required but increased the yield of as prepared activated carbon by 1.3 times and total organic carbon uptake from water per mass of utilized wood by 1.2 times.

## Supplementary Materials

The following are available online at www.mdpi.com/1996-1944/9/1/20/s1.
