# Supplementary material for: Activation of Aspen Wood with Carbon Dioxide and Phosphoric Acid for Removal of Total Organic Carbon from Oil Sands Produced Water: Increasing the Yield with Bio-Oil Recycling
Source: Materials (Basel). 2016 Jan 2;9(1):20. doi: 10.3390/ma9010020 (PMC5456554; doi:10.3390/ma9010020)
Supplement: Supplementary file 1 [file materials-09-00020-s001.pdf]

# Supplementary Materials: Activation of Aspen Wood with Carbon Dioxide and Phosphoric Acid for Removal of Total Organic Carbon from Oil Sands Produced Water: Increasing the Yield with Bio-Oil Recycling

**Andrei Veksha, Tazul I. Bhuiyan and Josephine M. Hill**

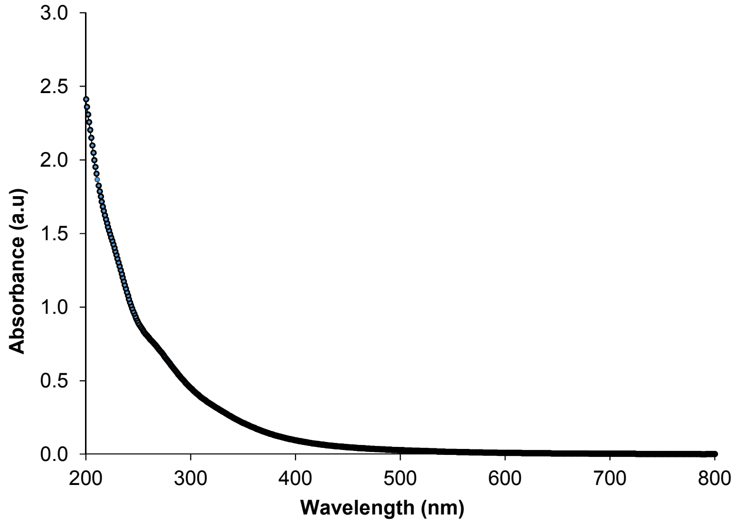


**Figure S1.** UV-vis spectrum of SAGD water after 25 times dilution with deionized water.

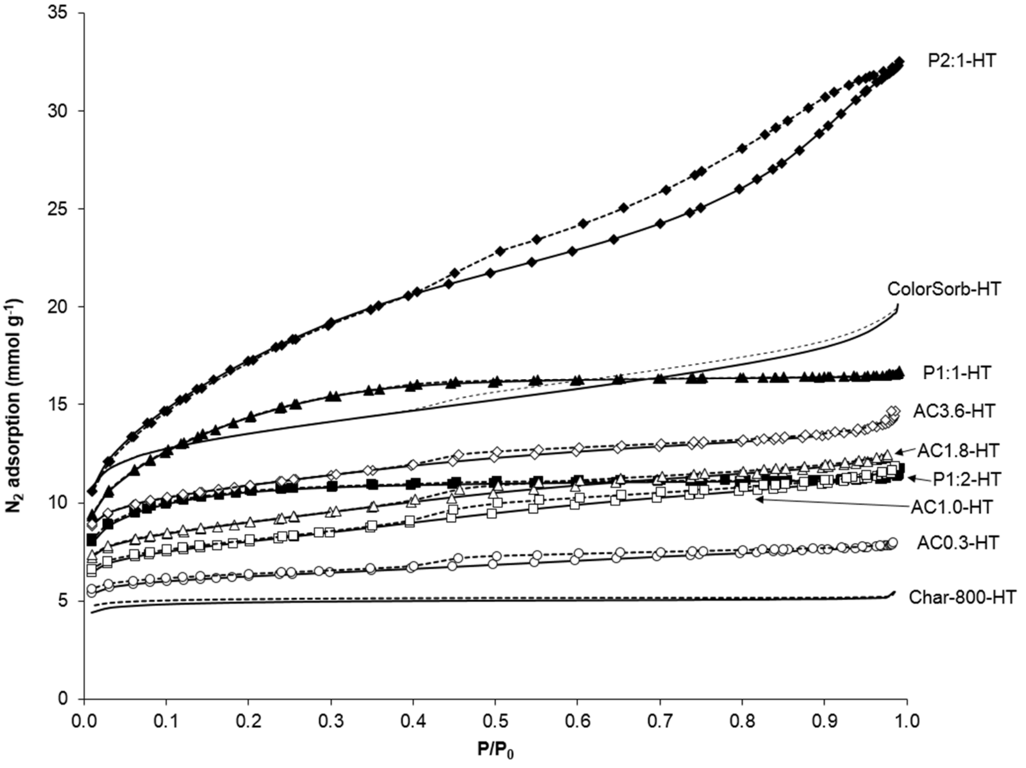


**Figure S2.** Selected $N_2$ adsorption (solid lines) and desorption (dotted lines) isotherms of the activated carbons.

Figure S2 shows adsorption isotherms of the non-activated char and activated carbons. The $N_2$ uptake by Char-800-HT exclusively at relative pressures $P/P_0$ below 0.1 indicates the highly microporous structure with narrow pore size distribution of this sample. $N_2$ adsorption capacities (isotherm plateau and nitrogen adsorption) of the $CO_2$ activated carbons increased with longer activation time. Evidence for the formation of micropores in these samples includes the increased $N_2$ uptake at $P/P_0$ below 0.1, while evidence for the formation of meso/macropores includes the lack of a plateau and a hysteresis loop. In $H_3PO_4$ activated carbon samples, the widening of pores and development of mesopores were enhanced by the higher $H_3PO_4$: wood ratio.

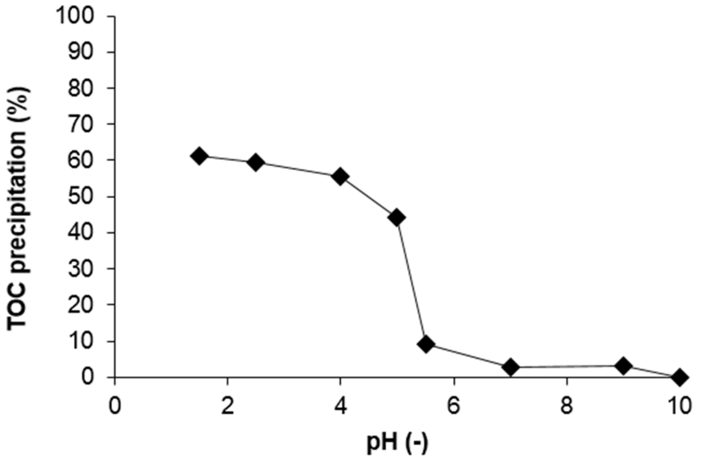


**Figure S3.** The influence of pH on the precipitation of TOC from SAGD water. The symbols correspond to the measurements, and the lines are only to guide the eye.

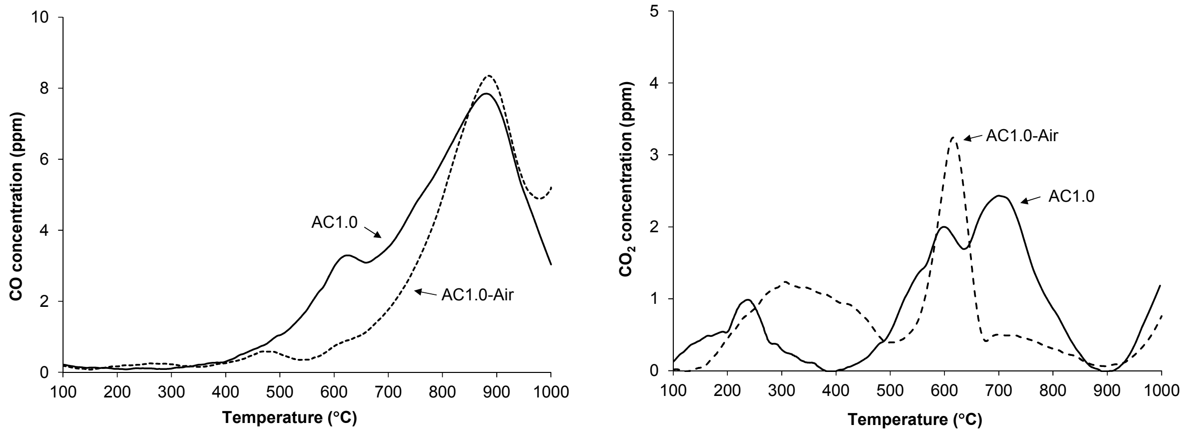


**Figure S4.** The CO (left) and $CO_2$ (right) TPD profiles of the carbon samples.
